# Hospital Meetings, &c.

**Published:** 1898-11-26

**Authors:** 


### HOSPITAL MEETINGS, &C.

### EAST LONDON HOSPITAL FOR CHILDREN.

#### Opening of the "Princess Mary" Convalescent Home, Bognor.

On Wednesday in last week took place the formal opening of the "Princess Mary" Home, at Bognor, convalescent branch of the East London Hospital for Children, Shadwell, E. The "top brick," so to speak, of a London hospital is emphatically its seaside or country home, and in no case is this necessary adjunct more urgently wanted than in connection with such a hospital as the East London, doing a splendid work in one of the most squalid and wretched districts of a dreary region.

A good many visitors came down from London for the opening ceremony, and a number of Bognor residents were also present. The Viscountess Falkland and Mr. Charles Cheston, upon whom should have devolved two of the principal parts in the day's proceedings, were both unfortunately prevented by illness from being present, and Lady Falkland's place was filled at the last moment by the Lady Maria Hood. Viscount Falkland, the members of the committee of management, Miss Rowe, matron of the Shadwell Hospital, and Miss Pinchard, matron of the new home, received the guests at the entrance, where Lady Maria was presented with a bouquet by a small patient, afterwards leading the way to one of the dormitories, where a short dedication service was read by the Rev. W. B. Bladon, of St. John the Baptist, Bognor. Lady Maria then declared the Home open for the reception of patients, and adjournment was made to the large "play-room" on the ground floor, where an excellent luncheon was prepared for the visitors. Toasts to the "Prosperity of the Home" and to "The Visitors" brought short speeches from Lord Falkland, the Earl of Erroll, and Dr. Conway Cooke, Lord Falkland appealing to all who wished the home well to aid in the work by contributing to its funds, and Lord Erroll speaking of its completion as a "culminating point in the history of the hospital," without which it was no exaggeration to say the work of the latter could not but be curtailed. The public were somewhat over-ready to rush with subscriptions to Lord Mayor's Funds for foreign objects, but charity after all began at home, and the care of our own children in England should come first A spell of fresh air was an everlasting benefit to the children of the poor, often suffering as much from want of nourishment as from disease.

The "Princess Mary" Home, named after the late Duchess of Teck, who was much interested in its establishment, has an attractive exterior. It is built of red brick, and roofed with red tiles; a special feature is the deep, sheltered balcony, running between the two gabled ends, over the front entrance, facing the sea and the south. The interior looked its best on Wednesday, and was bright with flowers. Miss Pinchard and her staff must be warmly congratulated on the result of their work, for they had but a bare ten days to settle in, arrange the furnishing, and take in a first batch of patients. To the right of the door as you enter comes first the matron's sitting room, and then two small wards with four cots in each, intended for those children who are not well enough to be up and dressed. At the back are lavatories and bath room. In the corresponding wing is an ideal play room, many-windowed, so that the sun shines into it all day long. The floor is parquet, and the walls are yellow with dark green dado, the same colouring being employed throughout the building. Behind the play room is the dining room, a buttery hatch connecting it with the kitchen. On the first floor are two large dormitories, each containing ten cots. Both face seawards, and are lofty and well ventilated. On the same floor, besides the lavatories and baths for the children, is the bath room and lavatory for the staff. A charming little sitting-room for the nurses opens on to the balcony, and next to that is the matron's bed-room. There is also a spare bed-room, intended for the use of a tired or convalescent nurse. The top floor is devoted to quarters for the nurses and servants. They are delightful rooms, most comfortably and prettily furnished, and all with a sea view. An excellent idea has been to fix below the windows, from one side of the room to the other, a bountiful arrangement of drawers and cupboards, acting also as dressing-table, while most of the rooms also boast a large hanging cupboard. The woodwork and the furniture throughout is of polished pine; the stairs of teak. The home is well equipped for its twenty-eight beds, but to complete the scheme, the total cost of which was estimated at £7,200, a laundry and small sanatorium have yet to be built.

There is no debt on the building, but more money is urgently wanted to make these two additions, and help in the maintenance, which will annually be at least £600. A visit to the wards at Shadwell, followed by a glimpse of their small occupants revelling in the fresh air and sunshine of Bognor, cannot fail to lead to the opening of hearts and purses for such a good purpose. There were eight children in the home on the opening day, white-faced little Londoners, looking very dainty in "Sunday best" white flannel, and very much appreciating their pleasant quarters. Presents of games and toys would, one may guess, be welcomed by Miss Pinchard, or contributions towards the maintenance of the wardrobe of little garments always needed for the children.

### LANTERN LECTURE AT ST. STEPHEN'S SCHOOLS, WESTBOURNE PARK.

An excellent lecture on the "Hospitals of London" was given by Sir Henry Burdett in the evening of the opening day of the new schools of St. Stephen's, Westbourne Park. There was a crowded attendance, and, as part of the audience were unable to see the lantern illustrations owing to the shape of the room, the vicar, Mr. Neligan, undertook to repeat it on another occasion for the benefit of those who were disappointed. Sir Henry Burdett began at the beginning of his subject, and briefly reviewed the magnitude of the work of the London hospitals, and the important bearing of that work upon everyone, from the Queen to the humblest labourer, man, woman, and child, in Her Majesty's dominions. He said that he purposed to take his audience from the out-patient department to the wards, thence to the operating theatre, to give them illustrations of operations in the olden days, then to one of modern date, to give them a glimpse of the work of the fever hospitals under the control of the Metropolitan Asylums Board and convalescent institutions. Passing thence to the practical and financial side of the matter, Sir Henry remarked that the people of London had not adequately grasped the fact that £100,000 a year were needed to put the hospitals on a sound basis. It was the object of the Prince of Wales's Hospital Fund to raise this money. The income of that Fund was now £35,000. It remained, therefore, with every individual to help to make up the balance of the £65,000 still needed. The issue of the hospital stamps enabled everyone, even children, to do this. By the purchase of an album and by affixing in the first two spaces stamps to the value of 1s. and 2s. 6d., the owner could have his photograph taken and inserted in his own album free. In the albums there was an autograph letter of the Princess of Wales, who has the success of the scheme very much at heart so fixed, by her own request, that it could not be removed. At a later interval Sir Henry said that it was of the utmost importance that the hospitals should be maintained by voluntary contributions; first, because in the rate-supported hospitals the public has to pay more than it ought or is necessary; and secondly, because experience all the world over shows that the standard of efficiency in State hospitals falls below that in the voluntary-supported institutions. He could attest this by personal observation, as he had visited many countries, and had been over hundreds of hospitals. It was right that the sick should receive the most perfect and highly-trained skilled attention that was procurable. The lantern slides were capital, and the lecture created much interest. Many purchasers came forward at the conclusion and bought albums and stamps from a lady who had kindly undertaken the sale. Slides of the different stamps were included in the display. The proceedings concluded with a hearty vote of thanks to the lecturer.

